# Fungal-Bacterial Interactions in Health and Disease

**DOI:** 10.3390/pathogens8020070

**Published:** 2019-05-21

**Authors:** Wibke Krüger, Sarah Vielreicher, Mario Kapitan, Ilse D. Jacobsen, Maria Joanna Niemiec

**Affiliations:** 1 Leibniz Institute for Natural Product Research and Infection Biology—Hans Knöll Institute, Jena 07745, Germany; wibke.krueger@leibniz-hki.de (W.K.); sarah.vielreicher@leibniz-hki.de (S.V.); mario.kapitan@leibniz-hki.de (M.K.); ilse.jacobsen@leibniz-hki.de (I.D.J.); 2 Center for Sepsis Control and Care, Jena 07747, Germany; 3 Institute of Microbiology, Friedrich Schiller University, Jena 07743, Germany

**Keywords:** mycobiome, microbiome, cross-kingdom interactions, polymicrobial, commensals, synergism, antagonism, mixed infections

## Abstract

Fungi and bacteria encounter each other in various niches of the human body. There, they interact directly with one another or indirectly via the host response. In both cases, interactions can affect host health and disease. In the present review, we summarized current knowledge on fungal-bacterial interactions during their commensal and pathogenic lifestyle. We focus on distinct mucosal niches: the oral cavity, lung, gut, and vagina. In addition, we describe interactions during bloodstream and wound infections and the possible consequences for the human host.

## 1. Introduction

### 1.1. Origins of Microbiota Research

Fungi and bacteria are found on all mucosal epithelial surfaces of the human body. After their discovery in the 19th century, for a long time the presence of microbes was thought to be associated mostly with disease. Only with an increased understanding of the microbial world and the increased use of antibacterial and antifungal drugs in the second half of the 20th century, people started to understand the beneficial role of microbes. Pioneer discoveries were, for instance, the production of vitamin B12 by intestinal bacteria or the protective effect of vaginal lactobacilli towards recurrent urinary tract infections (UTIs) [1,2].

The systematic in-depth analysis of bacterial communities living in different niches of the human body was accelerated by a rapid progress in sequencing techniques. In combination with bioinformatics tools, next-generation sequencing allowed for the identification of microbes that were previously uncultivable in the laboratory [3]. These advancements led to the “human microbiome project” which was initiated one and a half decades ago [4]. Soon afterwards, the first studies analyzing the complex fungal communities (termed “mycobiome”) were published [5,6].

Today, it is common knowledge that the human body contains as many microbes as the cells it consists of, and that the microbial communities are influenced by external and internal host factors such as food choices or genetics, respectively [7,8]. On the basis of this, contact and also interactions between bacterial and fungal members of the microbiota seem inevitable. The consequences of bacterial-fungal interactions for the human host are, however, largely unknown: What is the role of fungi in a healthy host? Are they a threat that needs to be controlled by the host and bacterial microbiota? Do some fungi have beneficial effects on the bacterial microbiota and the health of the host? Furthermore, in the context of dysbiosis and infections, does co-localization of fungi and bacteria affect the risk of infections?

### 1.2. Blooming Awareness of Cross-Kingdom Microbial Interactions

As pathogens, fungi were for a long time underdiagnosed and underestimated [9]. Especially fungi co-isolated with bacteria, were often considered irrelevant as they supposedly do not alter the outcome of the infection or stem from environmental contamination [9]. Again, improved diagnostics and increased awareness led to more studies reporting co-isolation of fungi and bacteria from patient material. Their results suggest that fungal-bacterial interactions frequently occur during infections. Herman et al. reported that among 68,000 clinical samples approximately 8% tested positive for *Candida* spp. and that the yeast was identified with and without accompanying bacteria [10]. Other studies reported that up to 38% of candidemia cases were mixed infections [11,12,13,14]. In cystic fibrosis (CF) patients, *Pseudomonas aeruginosa* is more frequently detected in individuals suffering from persistent *Aspergillus fumigatus* infection or persistent *Candida albicans* colonization than in patients without these fungi in their bronchoalveolar lavage cultures [15,16].

### 1.3. Micro- and Mycobiome Studies

There are two major experimental strategies used to understand fungal-bacterial interactions in humans and their impact on the host. First, entire microbial communities can be analyzed for the presence and relative abundance of certain species in the context of health and disease. While studies in humans are mostly correlative, animal models allow for the more specific manipulation of microbial communities. Secondly, fungal-bacterial interactions can be studied one-on-one with or without including the host. Here, the conclusions are more causative, but the number of interaction partners is limited.

The majority of studies published so far for both humans and animals, analyzed either the micro- or the mycobiome. To date, the number of investigations that analyzed both bacteria and fungi from the same sample or patient are relatively limited. However, those studies that were performed cover a broad range of diseases or treatment regimens, such as cancer, autoimmune diseases, cystic fibrosis (CF), or organ transplants [17,18,19,20,21,22,23,24,25,26]. These underlying conditions also represent risk factors for fungal and other opportunistic infections. Thereby, combined micro- and mycobiome analyses in these patients might be of special value to better understand if and how fungal-bacterial interactions affect the development of infections [17,18,19,20,27].

The simultaneous analysis of bacterial and fungal communities from one sample can be difficult as complete and unbiased cell disruption is required to retrieve nucleic acids for further analysis. Due to the rigid cell walls of fungi and some bacteria mechanical, chemical, or enzymatic lysis steps need to be included and tailored to the specific sample type in order to extract the nucleic acids [28,29]. In addition to the DNA isolation strategy, primer choice for amplification, sequencing approach, and the following taxonomical identification and their databases used are strongly dependent on the organisms of interest [30]. It should be mentioned that, to date, different studies used different approaches, and there is no gold standard protocol established for either bacteria or for fungi, let alone their combination [3,31]. Furthermore, common confounders of micro- and mycobiome analysis are variations between and within individuals, the difficult distinction between true colonizers and transient microbes in a certain niche, and the large proportion of uncultivable organisms in the laboratory. These technical obstacles lead to differences in the results from different studies but also explain inconsistent results obtained with culture-dependent and -independent methods [32].

Even though technical challenges remain in the analysis of complex microbial communities, it is generally accepted that interactions between the different members of the microbiota affect health and disease of their hosts [33,34,35,36,37,38,39,40,41,42,43]. 

### 1.4. Polymicrobial Interactions

All polymicrobial interactions can be classified as follows, no matter if they are exclusively bacterial, fungal, or cross-kingdom [33,41]. First, there is synergism, where one microbe creates a niche for another microbe to either colonize or infect. Secondly, during predisposition, one microbe interacts with the host and thereby predisposes it for colonization by the second microbe. Third, during microbial interference, the host interplay of the first microbe reduces or prevents colonization or infection with a second microbe. This reduction or prevention is also referred to as antagonism. Finally, if two otherwise non-pathogenic microbes cause disease only if combined, it is called addition [33,41].

**Figure 1:**
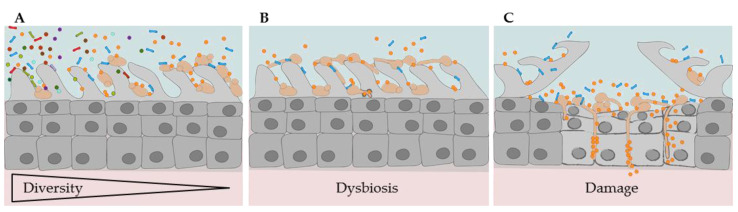
From dysbiosis to damage. Fungal-bacterial interactions can be beneficial or detrimental for the host. (**A**) High microbial diversity keeps individual fungal and bacterial species under control. Upon reduced diversity, due to, for example, antibiotics, certain species grow to increased abundance. (**B**) In low-diversity populations, opportunistic microbes switch from commensal to pathogen. (**C**) During co-infection, fungi and bacteria promote each other’s virulence, for example, by joined tissue penetration.

**Figure 2:**
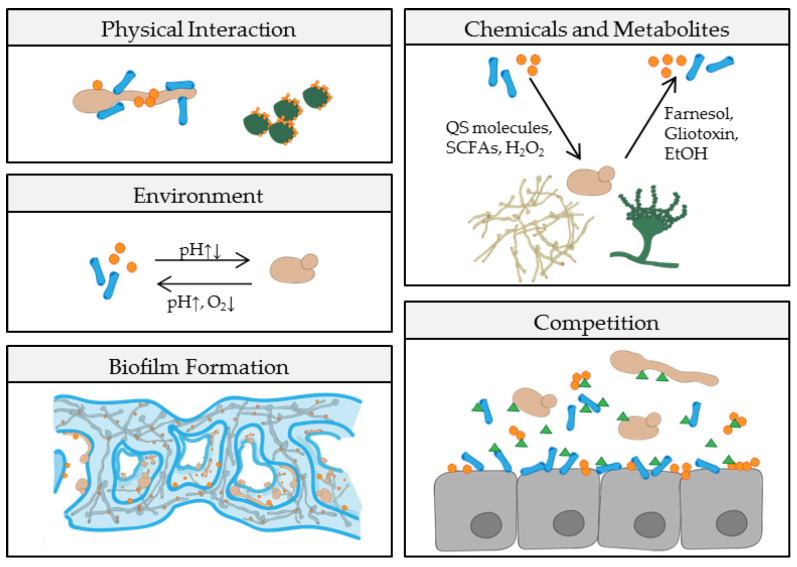
Modes of fungal-bacterial interaction. Fungi and bacteria interact via different modes of action. Direct binding leads to physical interaction. Release or consumption of chemical compounds, such as metabolic byproducts or quorum sensing molecules, mediates communication in a confined environment. Consumption of oxygen or release of protons influences the local milieu. Fungi and bacteria compete for nutrients or binding sites in a certain niche. Upon proliferation, mixed biofilms are assembled.

As implied by these four categories, fungal-bacterial interactions do not only impact the host. The host or the local environment can also impact the interplay of microbes, including fungi and bacteria. Nutrient supply, oxygen levels, and contact with the host immune system vary from niche to niche. In return, this influences the composition and behavior of the local microbial community. A switch from high-diversity homeostasis to low-diversity dysbiosis alters the metabolic status and virulence potential of many bacteria and fungi (Figure 1). Accordingly, different cross-kingdom combinations of microbial encounters are possible and with each combination the degree of antagonism or synergism changes. The underlying modes of fungal-bacterial interplay include physical or chemical interaction, modulation of environment or host, competition for nutrients or adhesion sites, and formation of mixed species biofilms (Figure 2, Table 1), all of which might vary among different niches in the host. While competition for the host adhesion site is more likely to have an antagonistic effect during colonization or infection, combined biofilm formation is more likely to affect the host negatively. Despite the increasing number of studies, it is not yet possible to reliably predict how a certain combination of bacterium and fungus will behave in a specific host niche. Furthermore, the mode of interaction in vitro is not always a clear predictor of the outcome in the human host. 

### 1.5. Scope of This Review

**Figure 3:**
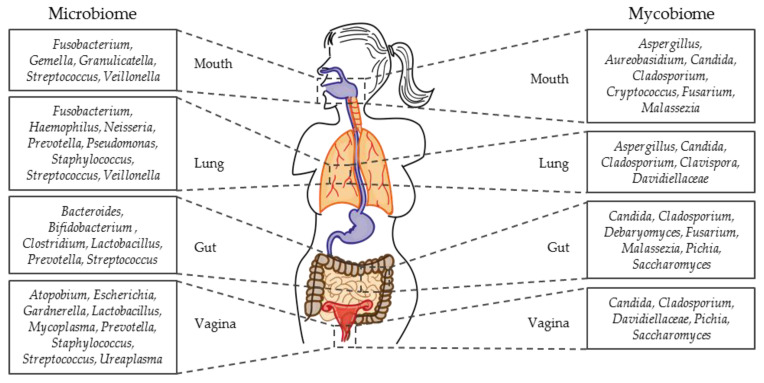
Microbial communities in mouth, lung, gut, and vagina of healthy humans. Most frequently identified bacterial (**left**) and fungal (**right**) genera are listed alphabetically and sorted by niche. Identification by culture and sequencing were considered [5,7,17,18,44,45,46,47,48,49,50,51,52,53,54].

In this review, we summarize the current knowledge regarding fungal-bacterial interactions in health and disease, during commensalism and infection. We introduce certain fungal-bacterial combinations in the niche where they are most relevant and studied. Of note, the combinations might be of medical relevance in more than one niche. We focus on the following four major mucosal niches naturally colonized with microbes: oral cavity, lung, gut, and vagina (Figure 3). In addition, we address the following interactions in niches that are sterile in healthy individuals: infections of medical devices, wounds, and bloodstream. Biofilms are included in the respective chapters if implied. Microbes of high medical relevance are briefly introduced in Box 1.

**Table 1:** Overview of modes of interplay and outcome of specific fungal-bacterial interactions. Interplay of fungi and bacteria occurs via multiple mechanisms and results in different outcomes for the host. Antagonistic relationships often limit microbial virulence and synergistic relationships potentiate pathogenesis. Listed below are combinations of fungi and bacteria that were investigated experimentally in vitro and in vivo for their effect on the host.

Mechanism	Fungi	Bacteria	Relationship	Study Setting	References
**Physical** **Interaction**	*Aspergillus* spp.	*K. pneumoniae*	antagonism	In vitro co-culture → prevention of spore germination and filamentation	[55]
	*A. fumigatus*	*P. aeruginosa*	antagonism	In vitro co-culture → decreased filamentation, biofilm formation, and conidia biomass	[56]
	*C. albicans*	*A. baumannii*	antagonism	In vitro co-culture → induced fungal apoptosis	[57]
		*F. nucleatum*	antagonism	In vitro co-culture → inhibited growth and filamentation	[58]
		Group B *Streptococcus*	synergism	In vitro: vaginal epithelial cells → enhanced fungal and bacterial adhesion	[59]
		*P. aeruginosa*	antagonism	In vitro co-cultures → killing of filamentous fungus	[60,61]
		*S. aureus*	synergism	Ex vivo mouse tongue infection [62]; in vivo oral mouse co-infection [63]; in vivo oral mouse infection [64] → promoted bacterial invasion	[62,63,64]
		*S. epidermidis*	non-competitive	In vitro adhesion model → bacteria bind to fungal germtubes	[65]
		*S. gordonii*	non-competitive	In vitro co-aggregation assays → *C. albicans* adhesin binds bacterial cell wall proteins	[66,67,68]
**Chemical** **Interaction and Release of Metabolic Byproducts**	*A. fumigatus*	*A. baumannii*	antagonism	Gliotoxin treated bacterial biofilm → decreased bacterial biomass	[69]
		*P. aeruginosa*	antagonism	In vitro co-culture → inhibited fungal biofilm formation [56]; Gliotoxin treated bacterial biofilm → decreased bacterial biomass [69]; In vitro assay → inhibited fungal growth [70]	[56,69,70]
		*S. aureus*	antagonism	Gliotoxin-treated bacterial biofilm → decreased bacterial biomass	[69]
	*C. albicans*	*A. actinomycetemcomitans*	antagonism	In vitro co-culture → AI-2 inhibits fungal biofilm formation	[71]
		*C. difficile*	antagonism	In vitro assay → p-cresol involved in filamentation	[72]
		*E. coli*	antagonism	In vitro biofilm assay → inhibited fungal biofilm formation [73]; In vitro assay → soluble factor kills *C. albicans* [74]	[73,74]
		*E. faecalis*	antagonism	In vitro biofilm model, in vivo nematode model, in vivo murine candidiasis model [75]; In vivo nematode model, in vitro biofiolm model [76] → inhibition of filamentation and fungal virulence	[75,76]
		*Lactobacillus* spp.	antagonism	In vitro: HeLa cells → reduced fungal adhesion [77]; In vitro *C. albicans* growth → stimulation of pseudohyphae and repression of growth [78]; In vitro model: vaginal epithelial cells → bactericidal mode against *C. albicans* [79]; In vitro co-culture → inhibition of filamentation [80]	[77,78,79,80]
		*P. aeruginosa*	antagonism	In vitro assay → inhibition of fungal growth [70]; In vitro co-culture → decreased bacterial virulence [81]; In vitro co-culture → reduces fungal viability [82]	[70,81,82]
		*S. aureus*	synergism	In vitro assay → enhanced tolerance to antimicrobial compounds	[83]
		*S. gordonii*	synergism	In vitro assay → enhanced filamentation	[68]
		*S. mutans*	synergism antagonism	In vitro assay → enhanced bacterial growth [84]; In vitro co-culture → inhibited filamentation [85,86]	[84] [85,86]
		*S. enterica* serovar Typhimurium	antagonism	In vivo nematode model, in vitro co-culture → repressed filamentation	[87]
	*C. neoformans*	*K. aerogenes*	synergism	In vitro co-culture → promoted fungal melanization	[88]
	*S. cerevisiae*	*Acinetobacter* spp.	synergism	In vitro co-culture, in vivo nematode model → enhanced bacterial growth and increased pathogenicity	[89]
**Influencing the Environment**	*C. albicans*	*B. fragilis*	synergism	In vitro assay → protection of bacteria by fungal biofilm	[90]
		*C. difficile*	synergism	In vitro co-culture → anaerobic growth of *C. difficile*	[72]
		*C. perfringens*	synergism	In vitro assay → protection by fungal biofilm	[90]
**Competition**	*C. albicans*	*Lactobacillus* spp.	antagonism	In vitro model: vaginal epithelial cells → reduced bacterial adherence	[79,91,92,93]
		*S. mitis*	antagonism	In vitro co-culture in a chemostat → competition for glucose	[94]
		*S. sobrinus*	antagonism	In vitro co-culture in a chemostat → competition for glucose	[94]
**Biofilm Formation**	*C. albicans*	*A. actinomycetemcomitans*	antagonism	In vitro Bioflux assay → decreased fungal biofilm formation	[71]
		*C. freundii*	non-competitive	In vitro co-culture → ability to form mixed biofilms	[95]
		*C. perfringens*	synergism	In vitro assay → protection by fungal biofilm	[90]
		*E. coli*	synergism	In vitro assay → increased mixed biofilm formation	[96]
		*E. faecalis*	synergism	In vitro assay → increased mixed biofilm formation	[97]
		*K. pneumoniae*	antagonism	In vitro assay → decreased fungal biofilm formation	[90,98]
		*P. aeruginosa*	antagonism	In vitro assay → decreased fungal biofilm formation	[60,99]
		*P. gingivalis*	synergism	In vitro assay → protection by fungal biofilm	[100]
		*S. aureus*	synergism	In vitro assay → increased mixed biofilm formation	[83,101,102,103]
		*S. epidermidis*	synergism	In vitro co-culture → increased mixed biofilm formation	[104,105]
		*Streptococcus* spp.	synergism	In vitro model: oral epithelial cells [106]; In vivo oral mouse model [107]; In vitro assay, in vivo oral rat model [108,109] → increased mixed biofilm formation	[106,107,108,109]
	*C. tropicalis*	*E. coli*	synergism	In vitro assay → increased mixed biofilm formation	[20]
		*S. marcescens*	synergism	In vitro assay → increased mixed biofilm formation	[20]
	*T. asahii*	*S. simulans*	non-competitive	In vitro co-culture → ability to form mixed biofilms	[95]

## 2. Fungal-Bacterial Interactions—Niche by Niche

### 2.1. Oral Cavity

#### 2.1.1. Niche Landscape of the Human Mouth

The oral cavity connects the outer world with the digestive tract and harbors one of the most diverse microbial communities in the human body [110]. On the basis of its anatomy, the mouth provides multiple niches that accommodate unique ecosystems for various microbes [111]. Saliva facilitates planktonic growth of microbes which do not stay in the mouth but get transported further into the stomach [112]. Secondly, the tongue, characterized by consistent shedding, is a mucosal surface where the majority of fungal biomass in the oral cavity is found [111]. Lastly, teeth, dentures, and oral implants provide a solid abiotic surface that provides a more stable base for microbes to form biofilms called dental plaque. These plaques can be divided further into supragingival plaque, located above the gum line, and subgingival plaque, located below the gum line [113]. At the supragingival and subgingival tooth surfaces, microbes find the most stable environment in the oral cavity [45].

Biofilms are three-dimensional growth forms of bacteria and fungi, combined and alone, that consist of cells as well as an extracellular matrix [114]. Biofilm formation is not only relevant on teeth, but also on medical devices such as implants or catheters and in wounds [114,115]. Biofilms are medically relevant because they serve as infection reservoirs for microbes [114,116,117]. Antimicrobial effectors, such as host immune cells or antibiotics, are less potent towards biofilm-associated bacteria and fungi as compared with planktonic microbes [118].

#### 2.1.2. Oral Micro- and Mycobiota in Health

Several studies analyzed the oral microbiome of healthy male and female adults. By using different next-generation sequencing (NGS) techniques, samples from various oral sites were combined to obtain the overall bacterial composition from all sub-niches. Of all identified taxa, 95% belonged to *Firmicutes*, *Proteobacteria*, *Actinobacteria*, *Bacteroidetes*, *Fusobacteria* and *Spirochaetes* [7,44,45,110,119]. Some genera such as *Streptococcus*, *Gemella*, *Granulicatella*, *Veillonella*, and *Fusobacterium* inhabited almost all oral subniches (Figure 3), whereas other genera, for example, *Prevotella*, *Bacteroides*, *Corynebacterium*, *Pasteurella*, and *Neisseria* were found in selected sites [7,44,45,119]. Compared to the bacterial composition, the fungal composition has been studied less often. In healthy individuals, the most frequent fungal genus was *Candida*, with *C. albicans* as dominant species. Other commonly identified fungi were *Cladosporium*, *Aureobasidium*, *Saccharomycetales*, *Aspergillus*, *Fusarium*, *Cryptococcus*, and *Malassezia* (Figure 3) [5,46]. In addition, several studies have analyzed not only the composition of the oral microbiota, but also its stability. By analyzing dental plaque, saliva, or tongue dorsum for longer periods some authors came to the conclusion that bacterial profiles were quite stable and that variations within individuals were smaller than between individuals [120,121,122,123,124,125]. On the contrary, other studies found that the oral bacteriome was variable and relative abundances could shift within periods as short as one day [126,127,128]. A study that analyzed the oral mycobiome stability over a period of 30 days revealed high interindividual diversity as seen for bacteria. But the frequency and abundance of different taxa was constant over time, indicating intraindividual stability [129].

#### 2.1.3. Oral Dysbiosis

Oral health is maintained by a complex equilibrium between different members of the resident microbiota. A shift towards dysbiosis, promoted, for instance, by dietary changes or antibiotics, reduces diversity and thereby enhances the risk of diseases like dental caries, or periodontitis [113]. Frequent intake of carbohydrates results in acidification of the local milieu due to sugar-fermenting, acid-producing bacteria [130]. The low pH provokes lesions in the tooth due to mineral loss which is the main feature of caries [131]. During caries progression, the continuously low pH in return also leads to changes in the microbiota and increased amounts of aciduric bacteria decrease the overall microbial diversity [131].

Oral candidiasis, or thrush, is on the other hand characterized by fungal overgrowth and invasion into superficial tissue layers, leading to damage of the oral mucosal surface [132,133]. Risk factors for oral *Candida* overgrowth include dysbiosis by bacterial depletion due to the use of broad-spectrum antibiotics, and also immunosuppression due to, for example, HIV infection [134]. Interestingly, thrush occurs also more frequently in newborns than in older children, suggesting that a stable oral microbiota and a mature immune system are important for controlling fungal growth [135,136,137,138,139].

Another oral disease associated with dysbiosis is gingivitis. It is an inflammatory disease, characterized by bacterial plaque that forms on tooth surfaces [113]. Without control, gingivitis can develop into chronic periodontitis. In this long-term inflammatory disease, destruction of periodontal tissue is induced by infiltration of immune cells. In turn, the tissue breakdown provides nutrients for bacteria and results in changes in the microbiota [140]. Several yeasts have been identified in the plaques of periodontitis patients using a range of different culturing techniques. Many samples were positive for *Candida* and *Rhodotorula*, but only *C. albicans* was found in all patients [141]. Furthermore, culturing experiments with oral rinse and periodontal pocket samples revealed higher rates of *C. albicans* in patients with severe periodontitis as compared to healthy controls [142], suggesting that the fungus might be involved in the development of this disease. *C. albicans* was also commonly detected in dental plaques of children suffering from early childhood caries [143,144,145,146].

#### 2.1.4. *C. albicans* and Bacteria in the Human Mouth

*C. albicans* is the fungus found most frequently in the oral cavity during dysbiotic disease, whereas *S. mutans*, a viridans group *Streptococcus*, was found to be the dominant bacterial species in dental plaque of many caries patients [147]. These Gram-positive, cariogenic bacteria are present in polymicrobial biofilms on the surface of teeth [148]. The pathogenicity of *S. mutans* is based on the formation of extracellular polysaccharides (EPS) by glucosyltransferases and their survival in acidic environments [148,149]. The exoenzyme glucosyltransferase binds to the surface of different microorganisms, which then accumulate and form adherent and cohesive biofilms known as dental plaque [148,150]. The presence of both *S. mutans* and *C. albicans* in early childhood caries dental plaques [143,144,145,146] suggests that interactions between these microbes might influence the disease. Indeed, in an in vivo model with female Sprague Dawley rats, Falsetta et al. observed enhanced biofilm-mediated virulence leading to rampant carious lesions in co-infected animals [109].

Furthermore, in vitro experiments with saliva-coated discs revealed *C. albicans-*induced expression of the *S. mutans* virulence gene glucosyltransferase B (GtfB) [109]. GtfB catalyzes the production of α-glucans and binds to mannans on the surface of *C. albicans* which promotes the extracellular matrix formation [151]. In addition, the experiments by Falsetta et al. identified EPS as the key mediator for mixed biofilm formation [109]. EPS was also shown to sequester the antifungal drug fluconazole in vitro which enhanced drug tolerance of *C. albicans* in the biofilm [108]. Furthermore, metabolic and chromatographic analyses of *S. mutans*- and *C. albicans*-derived conditioned medium revealed enhanced production of formate and farnesol. The fungal quorum sensing (QS) molecule farnesol has a known antibacterial effect at high concentrations, but it enhanced *S. mutans* cell growth and microcolony development in the dual-species biofilm with *C. albicans* [84].

Altogether, this illustrates a mutualistic relationship between *C. albicans* and *S.mutans* with synergistic contribution to virulent plaque formation in caries and exacerbation of disease severity. In contrast, Willems et al. argued that *C. albicans* might decrease *S. mutans* cariogenic potential by increasing the pH within mixed biofilms [152]. In an in vitro oral biofilm model, both microbes were grown on glass coverslips or hydroxyapatite disks to mimic the surface of teeth. After a few days, the pH was significantly higher in the mixed biofilm as compared with the bacterial single species biofilm. This alkalification could potentially prevent mineral loss in teeth [152], but this hypothesis has not yet been tested in experimental models. 

Other studies, however, do provide evidence for attenuating effects of *S. mutans* on *C. albicans* virulence. Injection of *S. mutans* cells or *S. mutans* culture filtrates into *C. albicans*-infected *Galleria mellonella* larvae increased survival rates of the animals [153]. Additionally, *C. albicans* showed reduced hyphae formation in the larval tissues when they were co-injected with *S. mutans* culture filtrates [153]. The antagonistic effect of *S. mutans* on *C. albicans* was mediated by the secretion of QS molecules. In vitro, the competence-stimulating peptide (CSP) and the fatty acid signaling molecule trans-2-decenoic acid were identified to inhibit germ-tube formation in *C. albicans*, which is an important virulence trait of the fungus [85,86].

Other *Streptococcus* species that are associated with oral candidiasis are *S. oralis*, *S. sanguinis*, and *S. gordonii*. They are also group viridans streptococci and are typical oral commensal bacteria. In contrast to *S. mutans*, these bacteria were shown to interact synergistically with *C. albicans* during infections, leading to exacerbated severity of oral candidiasis [106]. The presence of these streptococci enhanced the invasion of *C. albicans* through organotypic models of the oral mucosae under salivary flow conditions [106]. On the other hand, *C. albicans* promoted streptococcal biofilm formation on abiotic surfaces and on the surface of an oral mucosa analog in a flow cell system that mimics the oral environment [106]. Furthermore, a murine oral co-infection model confirmed enhanced colonization of *S. oralis* in the presence of *C. albicans.* While bacteria alone were not virulent, biofilm formation was increased in co-infections, and the frequency and size of oral thrush lesions were enhanced. Additionally, co-infection promoted deep organ dissemination of *C. albicans* [107].

The molecular basis for these interactions were, on the one hand, physical interactions mediated by Als adhesins on the yeast hyphae and antigen I/II family polypeptides on the bacterial surface [66,67,68]. In addition, secretion of the *S. gordonii* QS molecule autoinducer-2 (AI-2) enhanced hyphae development during co-incubations [68]. In oral epithelial cultures and in vivo in a murine model, co-infection increased the amount of proteolytic host protein µ-calpain that targets E-cadherin. That resulted in decreased E-cadherin levels in a culture model and triggered mucosal invasion and systemic invasion of *C. albicans* [154]. 

In addition to streptococci, several other oral bacteria were analyzed for their interplay with *C. albicans*. For example, the periodontal pathogens *Aggregatibacter actinomycetemcomitans* and *Fusobacterium nucleatum* inhibit germination of *C. albicans* in vitro by excretion of the QS molecule AI-2 or via physical interaction, respectively [58,71]. Co-cultivation of *C. albicans* and the periodontal pathogen *Porphyromonas gingivalis* under normoxia revealed increased viability of the bacteria in the presence of *C. albicans* biofilm, which suggested possible protection of the strict anaerobe *P. gingivalis* under aerobic conditions [100]. Additionally, pretreatment of oral gingival epithelial cells with heat-killed *C. albicans* resulted in enhanced invasion of *P. gingivalis*, indicating potential exacerbation of periodontal disease by the fungus [155]. 

Most aforementioned studies have analyzed the direct interplay of *C. albicans* with one bacterium. A recent publication by Janus et al., however, used a more complex approach. Here, a small oral microbiota was generated using pooled saliva samples as an inoculum for mixed biofilms with *C. albicans*. In these biofilms, *C. albicans* promoted the growth of anaerobic bacteria under aerobic conditions. Thereby, *C. albicans* affected the bacterial biofilm microbiome, indicating a role during oral microbiota homeostasis [156].

In summary, *Candida*-bacteria interactions in the oral cavity have been intensely studied, with both synergistic and antagonistic interactions observed. However, interactions of bacteria with other known fungal inhabitants of the oral cavity, such as *Cladosporium*, *Aureobasidium*, *Saccharomycetales*, *Aspergillus*, *Fusarium*, *Cryptococcus*, and *Malassezia* are not described yet, and the overall impact of fungal-bacterial interactions on oral health is only incompletely understood.

### 2.2. Vagina

#### 2.2.1. Niche Landscape of the Human Vagina

The composition of the vaginal micro- and mycobiota of healthy women is temporally dynamic. It changes related to menstruation, pregnancy, and health status. During reproductive years, high levels of glycogen and nutrients allow the colonization and dominance of anaerobic lactic acid producing lactobacilli, which contribute to a low pH (<4.5) [157,158,159,160].

#### 2.2.2. Vaginal Micro- and Mycobiota in Health

Drell et al. investigated the vaginal microbiome and mycobiome from asymptomatic, reproductive Estonian women [17]. They used the same samples taken from the vaginal fornix and cervix for the microbiome analysis as well as for the mycobiome analysis. The microbiome was determined by 16S rRNA sequencing and revealed lactobacilli as the most abundant bacteria. Other bacteria identified were *Gardnerella*, *Prevotella*, *Atopobium*, *Streptococcus*, *Ureaplasma*, *Escherichia*, *Mycoplasma*, and *Staphylococcus* (Figure 3). The vaginal mycobiome was studied by ITS1 sequencing. *Candida* was found to be the main genus, followed by *Saccharomycetales*, *Davidiellaceae*, *Cladosporium*, and *Pichia* (Figure 3) [17].

#### 2.2.3. *C. albicans* and Lactobacilli in the Human Vagina

Of all interactions described between fungi and bacteria in the vagina, *Candida* in combination with different bacterial strains is best described. *Candida* spp. are the causative agents of vulvovaginal candidiasis (VVC) in otherwise healthy women at childbearing age. About 75% of women suffer at least one episode of VVC in their life [161]. The main pathogen causing VVC in these women is the species *C. albicans*, followed by *C. glabrata*, *C. tropicalis*, and *C. parapsilosis* [161,162]. In addition to hormone replacement, pregnancy, immunosuppression, habits of hygiene, and other risk factors, the elimination of protective bacteria by antibiotic treatment also increases the risk for VVC [163]. As mentioned above, under normal conditions lactobacilli dominate the vaginal microbiota in healthy premenopausal women [164]. Lactobacilli are known to act antagonistically towards *C. albicans* by several mechanisms. As recently reviewed by Förster et al., these bacteria inhibit *C. albicans* by competition for nutrients and adhesion sites, inhibition of growth and hyphae formation, and excretion of fungicidal and fungistatic compounds [36]. 

Competition for adhesion sites is a typical mode of polymicrobial interplay with host cells. For *C. albicans* it was, for instance, demonstrated in vitro that its adhesion to vaginal epithelial or HeLa cells was reduced in the presence of different *Lactobacillus* strains [79,91,92,93]. This effect was not only mediated by the presence of the bacteria, but also by its supernatant alone, indicating that released compounds contribute to this protection [77]. In addition to adhesion sites, lactobacilli also compete with *C. albicans* for nutrients like glucose, as demonstrated in co-cultivation experiments [94]. 

Inhibition of the growth of *C. albicans* by lactobacilli was investigated in different studies. A bacteriocin-like peptide produced by a *L. pentosus* strain isolated from a prenatal woman induced pseudohyphae formation and inhibited growth of *C. albicans* when added to the growth medium [78]. The same was shown for bacteriocin-like compounds in the supernatant of several cultured *L. crispatus* and *L. jensenii* strains isolated from healthy premenopausal women [79]. Furthermore, Köhler et al. showed that *C. albicans* is sensitive to lactic acid at low pH [165]. Growth assays with lactic acid-containing MRS (De Man, Rogosa and Sharpe) broth revealed enhanced inhibition of growth, the lower the pH. They suggested that undissociated lactic acid permeates through the fungal plasma membrane at lower pH levels where it dissociates into protons and lactic acid counterions. The ions acidify the cytosol, interfere with cell metabolism, and thereby inhibit fungal growth [165].

Additionally, lactobacilli decreased *C. albicans* virulence by the inhibition of hyphae formation [80,166]. Filamentation was prevented by a low pH, which was generated by the production of different short chain fatty acids (SCFAs) by lactobacilli [80,167]. In spent culture media from *L. casei*, *L. rhamnosus*, or *L. paracasei* as well as in direct co-cultivation with *C. albicans*, excreted butyric acid inhibited germ-tube formation of *C. albicans* [80]. In an in vivo study with the nematode *Caenorhabditis elegans,* prophylactic provisions with *L. paracasei* inhibited *C. albicans* hyphae formation. This prevented cuticle rupture of *C. elegans* by *C. albicans* filamentation which otherwise killed the nematode [168]. Furthermore, SCFAs inhibited the enzyme histone deacetylase in *C. albicans* which impaired fungal growth and morphogenesis [166,169]. Also, pretreatment of *Galleria mellonella* larvae with *L. rhamnosus* increased survival after infection with *C. albicans* and led to decreased fungal CFUs [170,171,172].

In addition to directly affecting the fungus, lactobacilli also had an anti-inflammatory effect during *C. albicans* infection [173]. Pretreatment of HeLa cells with vaginal *L. plantarum* and *L. fermentum* isolated from healthy Cuban women reduced the production of proinflammatory cytokines IL-1β, IL-6, and IL-8 when challenged with *C. albicans* [93,173]. A decreased production of IL-8 was also shown in an in vitro HeLa cell model with *L. crispatos*, when cells were pretreated with the bacteria. Additionally, pretreatment induced the expression of human β-defensins and down-regulated expression of toll-like receptors (TLRs) on the cell surface [92,93]. Moreover, *C. albicans* grown on lactate as carbon source induced a different response in peripheral blood mononuclear cells (PBMCs) as compared to yeasts grown in glucose. In vitro, lactate-grown *C. albicans* increased the production of IL-10 and decreased the release of IL-17 by PBMCs. This makes lactate-grown *C. albicans* less perceivable to the immune system than glucose-grown *C. albicans* [174].

Because of their various antagonistic effects on different pathogens, lactobacilli are widely used as probiotics [175,176,177]. Delivery to the vaginal tract is enabled either directly by freeze-dried lactobacilli loaded on applicators, capsules, and tampons, or orally via the gastrointestinal tract (GI tract) by lactobacilli-containing capsules or food like yogurts [175,178]. Several studies showed an increase in lactobacilli numbers in healthy women after application of probiotics containing lactobacilli [179,180,181,182]. In VVC patients administration of probiotics increased the efficiency of azole treatment by reducing fungal colonization [183]. This led to a long-term cure and prevented relapse [183,184]. Probiotic treatment also improved the subjective resolution of symptoms like burning and itching [185].

#### 2.2.4. *C. albicans* and Streptococci in the Human Vagina

In the vagina, *C. albicans* interacts not only with lactobacilli. A synergistic relationship harmful for the host has been described between *C. albicans* and Group B streptococci (GBS) and *Escherichia coli* [186]. Both bacteria are associated with preterm birth, very low birth weight, and puerperal sepsis. *C.albicans* is described to be an independent risk factor for colonization with these bacteria [186]. Pidwill et al. demonstrated in an in vitro model with vaginal epithelial cells that GBS and *C. albicans* synergistically enhanced their capacity to associate with the host cells [59]. In vitro, adhesion was favored by physical contact of GBS and *C. albicans* hyphae via adhesins as shown using fluorescence microscopy [59]. Additionally, a mouse model of vaginal candidiasis by Yu et al. revealed increased fungal burden and levels of proinflammatory cytokines in a co-infection with GBS in vivo [187]. 

### 2.3. Gut

#### 2.3.1. Niche Landscape of the Human Gut

In the human body, the gut harbors the highest density of microbes which need to be contained within the gut lumen. At the same time, the gut is the organ facilitating the absorption of nutrients and water. Therefore, the gut mucosa has multiple functions which are reflected in its composition. The semipermeable barrier is built mainly by intestinal epithelial cells (enterocytes) that are complemented by mucus-producing goblet cells, paneth cells, and immune cells [188]. Enterocytes and paneth cells produce antimicrobial peptides that are constitutively secreted into the gut lumen. Interestingly, the majority of microbes resides in the mucus layer above the enterocytes and has no direct contact with the host. While most immune cells, including macrophages, plasma cells, und lymphocytes, are located in the lamina propria below the enterocytes, dendritic cells bridge the gap between gut lumen und lamina propria in order to sense them, distinguish between different microbes, and to either dampen or promote a subsequent immune response [189].

#### 2.3.2. Gut Micro- and Mycobiota in Health and Disease

The microbiota of the gut is probably the best studied microbial community of the human body. This can likely be explained by the high abundance of commensal microorganisms and the possibility to characterize the composition of bacteria and fungi through easily accessible feces [190,191]. The intestinal microbiota within one individual is highly dynamic due to the permanent exposure to the outside environment and fluctuation of internal factors. Diet, gender, age, and medication can shape the resident microbial community [192,193,194,195]. Within the gut environmental parameters like oxygen levels, pH, or the availability of macro- and micronutrients vary, which influences the diversity and density of bacteria and fungi [196]. 

The four dominating bacterial phyla in the gut microbiota of healthy individuals are *Bacteroidetes*, *Firmicutes*, *Proteobacteria*, and *Actinobacteria* (Figure 3) [197,198,199]. Especially the genera *Bacteroides*, *Clostridium*, *Prevotella*, and *Streptococcus* are detected [51,52]. 

As indicated previously, investigating the gut microbiome using feces is practical and easy. Nevertheless, different studies pointed out that the fecal microbiota differs from the mucosal microbiota [200,201,202]. Fecal samples are more representative of the luminal microbial community than of the mucosal community [52,203]. For a complete characterization of the intestinal microbiome, mucosal and luminal/fecal microbiota analyses should be combined but this is not feasible in most cases. Mucosal microbiota can only be examined in tissue samples from intestinal biopsies or surgery [204]. While these procedures are commonly performed in patients suffering from inflammatory bowel disease (IBD), this is not the case in healthy individuals [201,205,206,207]. As demonstrated by Conte et al., IBD altered not only the microbiota in feces, but also in the mucosal community. They found a higher abundance of mucosa-associated aerobic and facultative anaerobic bacteria in IBD patients as compared with the control participants [208].

The role of commensal intestinal bacteria in influencing human health is well accepted [209,210,211]. They are involved in different metabolic functions, interact with the immune system, and play an important role in energy harvest and storage [212]. In comparison, relatively little is known about the gut mycobiota. In the past, studies focused on the examination of bacteria due to the superior number of bacteria compared to fungi as well as technical limitations [213]. The rising incidence of mycoses and their origin from members of the microbial community has however raised interest in the mycobiota. Today, it is estimated that at least 0.1% of all 10^14^ microorganisms in the human gut are fungi [198,214].

Defining the gut mycobiota from healthy stool identified *Ascomycota* and *Basidiomycota* as the most abundant taxa, and the dominant genera are *Saccharomyces*, *Candida*, *Malassezia*, and *Cladosporium* [53,54,205]. Through examinations of healthy mice, it could be demonstrated that the most abundant genera of fungi in the gut of mice are also present in humans [27]. The fact that only a few of these fungi are contained in the food of mice suggests that the majority of the fungi in mouse intestines are indigenous [6,27].

As mentioned above, to date, the majority of studies have analyzed the abundance and composition of the bacterial community in the human and murine gut. However, a growing number of studies have considered both the bacterial and fungal compositions, which were investigated from the same sample [20,24,25,27,215,216,217,218,219]. Some of these studies were conducted to get insights into certain disease states in patients. For instance, Hoarau et al. showed a close association between endogenous gut bacteria and fungi in Crohn’s disease (CD) patients with a correlative increase in potentially pathogenic bacteria and the fungus *C. tropicalis*, and a decrease in beneficial bacteria. Furthermore, Sokol et al. investigated the role of bacterial and fungal components of the fecal microbiota and observed an imbalance in the *Basidiomycota*/*Ascomycota* ratio in IBD as compared with healthy subjects. Chakravarth et al. demonstrated dysbiosis in the intestinal micro- and mycobiota of Keratitis patients and a higher abundance of bacteria, for example, *Bacteroides fragilis*.

A growing body of evidence suggests that the fungal community is an important factor for human health and disease. An example of this is the study by Wheeler et al., which demonstrated that the normal gut mycobiota with members like *Malassezia* and *C. albicans* have a protective role for the host [220]. Treatment of mice with the antifungal drug fluconazole caused dysbiosis in the gut, and as a consequence, the level of opportunistic fungi like *Aspergillus amstelodami*, *Epicoccum nigrum*, and *Wallemia sebi* increased. This led to elevated disease severity in the mouse models of acute or chronic colitis [220]. In line with this finding, a recent study investigated the protective role of fungi against tissue damage during colitis and viral infection. The authors demonstrated that upon reduction of the microbiota, mono-colonization with *S. cerevisiae*, *C. albicans,* or fungal cell wall mannans were sufficient to mitigate harmful effects [221]. Further, different studies reported that dysbiosis of the mycobiota correlated with the onset of CD [20,205,215].

Although the mycobiota can have positive effects on the host, the increased fungal burden is also a risk factor for disseminated candidiasis [27,222]. Long-term and/or broad-spectrum antibiotic treatment is one of the major risk factors for disseminated candidiasis originating from the gut because it depletes bacteria that restrict fungal overgrowth [27,222,223]. 

#### 2.3.3. *Candida* spp. and Bacteria in the Human Gut

The crucial role of intestinal bacteria to mediate colonization resistance against *C. albicans* has been demostrated in mice, where antibiotic-induced disruption of the resident microbial communities led to stable expansion of the fungus [27,222,224]. Consistent with this, current murine research models used germ-free mice or mice treated with antibiotics to facilitate easy colonization with *Candida* species [225,226,227]. Further, it was shown that the bacterial microbiota in mice changed due to *C. albicans* colonization, characterized by a decrease of *Bacteroidetes* and *Synergistetes*, while *Firmicutes* stayed stable [228]. The presence of *Firmicutes* and *Bacteroides* seems important to maintain *C. albicans* colonization resistance in mice. These resident bacteria were shown to activate the transcription factor HIF-1α in intestinal cells, which led to an increased production of the antimicrobial peptide LL-37. LL-37, a cathelicidin, has anti-*Candida* activity and was shown to decrease *C. albicans* colonization in mice [226,229]. Recent studies by Charlet et al. investigated fungal-bacterial community variation in a mouse model of DSS-induced colitis. In mice inoculated with *C. glabrata*, DSS-treatment promoted overgrowth of these fungi, which in return worsened inflammation. It also led to an increased abundance of *E. coli*, *E. faecalis*, and *Bacteroides vulgatus*, while *Lactobacillus johnsonii*, *Bacteroides thetaiotaomicron*, and *Bifidobacterium* decreased [230]. In the same model, additional oral administration of β-glucan decreased aerobic bacteria and IL-1β expression but increased *L. johnsonii*, *B. thetaiotaomicron*, and IL-10 production promoting the elimination of *C. glabrata* [231].

#### 2.3.4. *Saccharomyces* spp. and Bacteria in the Human Gut

It is not only the entire intestinal microbial community that can control opportunistic pathogens, but also the interplay between single bacteria and fungi might be of clinical relevance. A well-studied example is baker’s yeast *Saccharomyces cerevisiae*. The dimorphic yeast *S. cerevisiae* is widely distributed in the environment and commonly used in the food industry for the production of baked goods or alcoholic beverages [232]. *S. cerevisiae* is one of the best characterized fungal organisms and used as model eukaryote in research. Still, there is also evidence that this yeast can occasionally cause superficial and systemic infections [233,234,235]. The related strain *Saccharomyces cerevisiae boulardii* is used clinically as a probiotic and as a preventive measure against *Clostridium difficile* infection [236,237]. Jiang et al. documented a protective role of *S. cerevisiae* in mice against inflammatory disorders by the replacement of enteric bacteria [221]. Furthermore, *S. cerevisiae* was shown to be able to reduce intestinal translocation of enterotoxigenic *E. coli* (ETEC) and modulate the mucosal immunity in pigs [238]. A more recent study confirmed this and provided additional insights into the interplay of *E. coli* and *S. cerevisiae* by using in vivo and in vitro approaches such as intestinal cell lines, co-culture assays, and antibiotic-treated mice: Roussel et al. showed that *S. cerevisiae* reduced bacterial growth, decreased bacterial colonization, and inhibited the adhesion of ETEC [239]. This implies that *S. cerevisiae* could be used as a potential probiotic to control ETEC infections. Interestingly, the *Enterobacteriaceae Salmonella enterica* serovar Typhimurium inhibits hypha formation by the Type III secretion system effector SopB, and thereby reduces *Candida* virulence in *C. elegans* [87].

#### 2.3.5. Yeasts and *Clostridia* spp. in the Human Gut

As aforementioned, probiotics are used to counteract colonization and infections with *C. difficile*. Infection with this Gram-positive bacterium often occurs after antibiotic treatment and can manifest as diarrhea, colitis, sepsis, and, in fatal cases, death [240]. Fecal matter transplants (FMT) that reconstitute the normal microbiota are the only effective treatment against *C. difficile* infections. To this date, the optimal and most beneficial composition of such a transplant is yet to be determined. As mentioned above, Massot et al. described that *S. cerevisiae boulardii* reduced *C. difficile* growth in hamsters [236]. The potential beneficial role of this fungus was also implied by studies in mice that successfully used *S. cerevisiae boulardii* to reduce *C. difficile*-induced mortality [241]. 

In contrast, the impact of *C. albicans* on *C. difficile* is less clear. Co-culture studies have shown that *C. albicans* helps obligate anaerobic *C. difficile* and *C. perfringens* to grow under aerobic conditions. This could explain the observation of a study in mice that found that a high abundance of *C. albicans* in stool samples correlated with reduced efficacy of FMT [242]. The fact that the presence of *C. albicans* worsens the disease severity of *C. difficile* infection in the mouse model was also demonstrated and expressed in a reduced survival rate and enhanced generalized bowel edema in vivo [243]. In contrast, a study by Markey et al. described that *C. albicans* could interact antagonistically with *C. difficile* in a mouse model [244]. *C. albicans* colonization led to increased cytokine IL-17A levels upon *C. difficile* infection and reduced mortality [244]. In this study, the fungus did not mediate colonization resistance towards *C. difficile*, but it altered the bacterial microbiota, and therefore it appears possible that the effect of *C. albicans* on *C. difficile* infection depends on the overall microbiota composition. 

#### 2.3.6. *C. albicans* and *E. faecalis* in the Human Gut

Another bacterium commonly found in the human large intestine, and thus inhabiting the same niche as *C. albicans*, is *Enterococcus faecalis* [34]. Both, in vitro and in vivo studies in the model organism *C. elegans* demonstrated that *E. faecalis* acted antagonistically towards *C. albicans* and decreased its virulence by negatively influencing filamentation [76]. The *E. faecalis* bacteriocin EntV was identified as the key mediator of hyphae inhibition [75]. Interestingly, this contradicts previous results, which described synergism between *C. albicans* and *E. faecalis* during biofilm formation on endotracheal tubes in ventilator-associated pneumonia patients, and promotion of *E. faecalis* colonization in the mouse cecum after antibiotic treatment with cefoperazone [97]. 

### 2.4. Lung

#### 2.4.1. Niche Landscape of the Human Lung

For a long time, it was assumed that healthy lungs are sterile and that the presence of microbes indicates an infection [245]. Today, improved culturing techniques and culture-independent methods convey a different message, which is that a complex microbial community colonizes the lung [49,50]. In contrast to most other niches, the lung is consistently exposed to air, which results in a uniquely composed microbiota, while the total microbial biomass is relatively low [190]. Common with the gut, the lung is not a uniform tissue and provides different habitats with multiple growth conditions, such as variations in the pH level, temperature, and oxygen level [246,247]. Furthermore, a wide range of cells that are involved in the immune responses are present in the lung. This includes epithelial cells with the ability to release antimicrobial peptides, memory T cells, and resident macrophages [248].

#### 2.4.2. Lung Micro- and Mycobiota in Health and Disease

The most commonly isolated fungal genera from bronchoalveolar lavage cultures of healthy volunteers are *Aspergillus*, *Candida*, *Penicillium*, *Clavispora*, and *Davidiellaceae* (Figure 3) [18,50]. Also, *Pneumocystis* are often identified in immunocompromised patients [249,250,251], where they can cause pneumonia [252,253]. Studies also reported the carriage of *Pneumocystis* in immunocompetent, healthy volunteers in 20% of a Spanish cohort and up to 79% in Santiago, Chile [253,254,255]. *Pneumocystis* is strongly adapted to life in its host, and as of today no in vitro laboratory culture system for *Pneumocystis* has been described [256,257]. Therefore, if and how *Pneumocystis* might interact with bacterial lung microbiota is still unknown.

The core bacterial microbiota in healthy lungs contains the genera *Streptococcus*, *Pseudomonas*, *Prevotella*, *Veillonella*, *Haemophilus*, *Neisseria*, and *Fusobacterium* (Figure 3) [47,48,49]. To get a deeper insight into this unique body site, programs like the Lung HIV Microbiome Project investigated volunteers infected with HIV in relation to healthy controls with regard to the microbial community and disease status [258,259,260]. Although the process of characterization and understanding of the lung microbial community is still in its infancy, it is well accepted that the respiratory microbiota has an impact on the preservation of lung health and manifestation of acute or chronic respiratory disease [213,261,262,263]. 

Current research focuses mainly on microbial dysbiosis during chronic lung disorders, such as chronic obstructive pulmonary disease (COPD), CF, or asthma [248,262]. One of the best-studied patient groups, regarding the interplay of bacteria and fungi and their impact on disease severity, is the group of patients suffering from CF [245]. In their lungs, the mucus is thicker and more rigid which results in an ideal reservoir for many microbes. Oxygen levels in the mucus vary from high to low. Antimicrobial agents or immune cells can penetrate the mucus less efficiently [264]. These conditions support simultaneous co-colonization with more than one pathogen, as often documented in CF patients [39,265,266]. Especially co-colonization with two potentially pathogenic species represents a risk factor for CF patients and is associated with a higher mortality and morbidity rate [42]. The most commonly found bacteria in CF patients are *P. aeruginosa* and *S. aureus*, while *A. fumigatus* and *C. albicans* are the most frequently isolated fungi [256]. 

#### 2.4.3. Fungi and *P. aeruginosa* in the Human Lung

The Gram-negative bacterium *P. aeruginosa* is often co-isolated with different human opportunistic fungi in CF patients [56,267,268,269], and a common cause of infections in these patients [39]. Its interactions with different fungi have been studied in great detail a prime example is the interaction with the mold *A. fumigatus*. As one of the first studies investigating cross-kingdom polymicrobial interactions, it was shown already in 1999 that pyocyanin and phenazine, compounds secreted by *P. aeruginosa*, inhibit fungal growth of *A. fumigatus* and *C. albicans* in vitro [70]. Investigations in a co-culture assay revealed growth inhibition of *A. fumigatus* that was recapitulated by the volatile compound dimethyl sulfide over distance [270]. Mowat et al. demonstrated that direct contact of *P. aeruginosa* and *A. fumigatus* in co-culture led to the release of diffusible extracellular molecules, which decreased fungal filamentation, biofilm formation, and conidia biomass [56]. It was also shown that already existing fungal biofilms were more resistant towards the inhibition by *P. aeruginosa* than conidia or germlings. Of note, these effects were depending on the isolation source and growth phenotype of the bacteria [271]. In return, *Aspergillus* inhibited biofilm as well as single cell growth of *P. aeruginosa* in vitro, [69]. Furthermore, hyphae of *A. fumigatus* produced gliotoxin, which had a general anti-biofilm effect on different bacteria, such as *P. aeruginosa* as well as *S. aureus*, and *A. baumannii* [69]. 

Furthermore, *P. aeruginosa* reduces growth and filamentation of other fungi commonly isolated from the lung of CF patients, such as *Cryptococcus neoformans*, *C. albicans*, and *Scedosporium aurantiacum* [267,269,272,273]. In vitro cultures showed that the growth of the filamentous fungus *S. aurantiacum* was inhibited by *P. aeruginosa*, especially the formation of hyphae [274]. Interestingly, the involvement of pyocyanin and phenazine could be excluded as the key effectors during inhibition of *S. aurantiacum*, while other studies continued to link the two molecules to the reduction of hyphae formation [272]. Although the exact mechanism for the antagonism between *S. aurantiacum* and *P. aeruginosa* is uncertain, the authors suggested that small molecules could be responsible for the inhibition [274].

As described above, many of the fungal-bacterial interactions are mediated by the secretion of small molecules. However, investigations of *Candida–Pseudomonas* interactions also showed the importance of direct contact [61]. *P aeruginosa* attached in vitro directly to the hyphae of *C. albicans*, formed biofilms, and secreted phenazine, which led to death of the filament. Interestingly, this only affects *C. albicans* hyphaeand not yeasts [60]. The authors propose that destroying hyphae enables the bacterium to obtain nutrients from *C. albicans* in a biofilm. 

Furthermore, it is well described that the secretion of farnesol produced by *C. albicans* alters *P. aeruginosa* QS. Addition of farnesol decreased the production of *Pseudomonas* quinolone signal (PQS) which correlates with the secretion of pyocyanin [81]. Remarkably, the swarming behavior of *P. aeruginosa* is also altered by farnesol which could lead to the formation of a higher biofilm biomass [60]. It is widely accepted that observations in vitro do not necessarily translate into in vivo models or even human patients. With increasing complexity, more parameters influence the microbes analyzed. The comparison of the interactions between *P. aeruginosa* and *C. albicans* that was mentioned above illustrates this. While the fungus and the bacterium, acted antagonistically in in vitro co-cultures, in vivo investigations in rats demonstrated a synergistic collaboration between the two pathogens [40,70,269,275]. Further, tt could also be shown in CF patients, that co-colonization with a pathogenic fungus, such as *C. albicans*, and *P. aeruginosa* is associated with a deterioration of health and an aggravation of the clinical outcome [39]. 

Additionally, results of different in vivo studies in mice were contradictory. Ader et al. reported decreased lung inflammation and number of *P. aeruginosa* cells after the administration of *C. albicans* to mice. In contrast, Roux et al. showed that *C. albicans* colonization of murine airways facilitated the development of bacterial pneumonia with *P. aeruginosa*, *E. coli*, or *S. aureus* by inhibition of phagocytosis of alveolar macrophages [275,276]. In human patients, two studies reported that *Candida* spp. colonization in patients with bacterial ventilator-associated pneumonia increased morbidity and prolonged the stay in hospital [277,278]. However, more research on the subject is needed to improve the clinical implications of pulmonary *Candida* colonization. 

#### 2.4.4. Fungi and *Klebsiella* spp. in the Human Lung

Another bacterial pathogen that causes ventilator-associated pneumonia is *Klebsiella pneumoniae*. *Klebsiella* spp. were detected in about 8.4% of the samples from ventilator-associated pneumonia patients in a US database [279]. The Gram-negative bacterium inhabits the lung and the intestine of the human body and is especially troublesome for immunocompromised patients in hospitals due to the occurrence of multidrug-resistant strains [53,280,281]. The antimicrobial resistance in based onthe presence of plasmids with resistance genes and their ability to form biofilms and capsular polysaccharides [282,283,284]. Nogueira et al. investigated interactions between this bacterium and different *Aspergillus* species using co-culture experiments [55]. They showed that *K. pneumoniae* could inhibit spore germination, hyphal growth, and biofilm formation of several *Aspergillus* species, such as *A. fumigatus*, *A. terreus*, *A. niger,* and *A. flavus* in vitro. The study also showed the importance of physical contact and the presence of live bacteria for the inhibitory effects [55]. A similar antagonistic effect of *K. pneumoniae* on the thickness of *C. albicans* biofilms was observed by Fox et al. [90]. In contrast, the closely-related bacterium *K. aerogenes* was shown to have a synergistic relationship with *C. neoformans* in co-cultures [88]. This fungus is typically surrounded by a polysaccharide capsule and colonizes a variety of environmental niches. In co-cultures, *C. neoformans* benefited from a substrate produced by the bacterium, which promoted the melanization of the fungus and thereby enhanced the resistance to external factors [88]. 

In addition to the aforementioned bacteria, the Gram-negative bacterium *Acinetobacter baumannii* is a bacterial pathogen with in the lung,. Diseases caused by *A. baumannii* range from pneumonia and meningitis to sepsis, are difficult to treat and frequently associated with high morbidity due to the occurrence of multidrug resistance [285]. Often, *A. baumannii* can be isolated from oral biofilms, which are the main reservoir for the emergence of COPD or pneumoniae [286]. Fungal-bacterial co-culture experiments demonstrated that the presence of *S. cerevisiae* leads to enhanced growth of *A. baumannii*, *A. haemolyticus*, *A. johnsonii*, and *A. radioresistens*. Here, ethanol was identified as the diffusible factor to cause this effect [89]. In contrast to this synergistic effect, outer membrane protein A (OmpA)-mediated attachment of *A. baumannii* to *C. albicans* filaments induced fungal apoptosis in co-culture [57]. In the context of the lung, however, in vivo experiments in rats indicated that pre-colonization with *C.albicans* facilitated the emergence of *A. baumannii* pneumonia with heavier lungs and a higher CFU burden than in control animals. Finally, a modulation in the expression of *A baumannii* virulence genes was detected [287].

#### 2.4.5. Mucorales and Bacteria in the Human Lung

Similar to Aspergilli, molds of the order *Mucorales* can infect immunocompromised humans via ubiquitous spores that eventually form filaments in the target organ. *Mucorales* infects the lungs, but also other cavities of the upper respiratory tract, wounds, the GI tract, and the bloodstream [288]. The most common genera associated with mucormycosis are *Rhizopus*, *Rhizomucor*, and *Lichtheimia* [289,290]. In the environment, but also in patients, *Mucorales* interacts inevitably with various bacteria. Gram-negative *Serratia marcescens* was demonstrated to migrate on hyphae and kill them by a yet undefined mechanism [291]. Remarkably, many of the *Mucorales* spp. were shown to harbor endosymbiotic bacteria of different species [292,293]. For example, the genus *Rhizopus* was shown to harbor endosymbiotic *Burkholderia* which produces the plant mycotoxin rhixozin. When tested for its impact on fungal virulence, the ability of *Rhizopus* to induce endothelial cell injury in vitro did however not differ between strains with or without an endosymbiont. Furthermore, eradication of the bacteria did not decrease virulence of the fungi in a diabetic mouse model or in fruit flies [292].

### 2.5. Wound, Medical Device-Associated, and Systemic Infections

#### 2.5.1. Niche Landscapes of Skin, Wound, and Bloodstream

Fungal-bacterial interactions occur in body sites naturally colonized with multitudes of microbes, but also in niches that are considered sterile in the healthy host (i.e., blood, tissue, and also medical devices). In these, the number of interacting and competing microbes is often more limited, but the requirements towards the microbes dictated by the host differ also from mucosal surfaces. This likely affects the nature of the fungal-bacterial interplay. In wounds and on medical devices, mixed biofilms are of high medical relevance. 

Microbial colonizers, as well as microbes temporarily residing on the skin, can reach into underlying tissue through micro- and macrotrauma, for example, cuts and burns [294]. The progression of the infection then depends on the host immune status and measures of treatment [294,295]. In all cases, the transfer from the skin into a wound, and from there possibly into the bloodstream, is accompanied by several changes of the physiological environment which affect the microbes’ metabolism, virulence, and possible encounter of other microbes. 

Healthy human skin is composed of two major layers which are the epidermis and the dermis. The surface of the epidermis is dry, slightly acidic (pH 5.5), scarce in nutrients, and colder than the underlying tissue [295]. In addition to the bare conditions of the epidermis, sweat glands produce salty sweat, antimicrobial fatty acids and peptides, and lipid-rich sebum [295]. In contrast to intact skin, the wound is a much more complex environment. In addition to the humoral effectors present in the skin, as for example antimicrobial peptides, immune cells infiltrate from the underlying tissue into the wound. Within the wound or abscess, nutrient and oxygen availability can be reduced and microbial competition may be increased [295]. Just as on medical devices, biofilm formation is involved in the pathogenesis of wound infections [95,294]. In blood, microbes are challenged by immune cells and the complement system andthey also have to cope with body temperature, high glucose and low micronutrient concentrations, as well as physical forces due to blood flow [296].

#### 2.5.2. Micro- and Mycobiota of Wounds

Most common colonizers of the human skin are *Propionibacterium* spp., *Staphylococcus* spp., *Corynebacterium* spp., and the fungus *Malassezia* [295]. These skin commensals are also dominating fresh wounds, while chronic non-healing wounds and slowly-healing burns are more often colonized by members of the gastrointestinal flora or the respiratory tract [294]. This is reflected in two key studies that analyzed fungal-bacterial composition and interplay in wounds, the formation of mixed biofilms and impaired wound healing. First, Kalan et al. sequenced samples of 100 non-healing foot ulcers of diabetic patients and detected that 80% contained fungal DNA. In contrast, only 5% of these samples were positive for fungi when analyzed by culture. The fungal biomes were very heterogeneous and influenced by the administration of antibiotics. Nevertheless, the two most commonly identified species were the environmental mold, *Cladosporium herbarum,* and the yeast *C. albicans*. The presence of *C. albicans* correlated with higher inflammation, necrosis, and longer healing times. When ulcer samples were cultured in vitro, they formed mixed biofilms with yeasts and bacteria. As examples, Kalan et al. tested biofilms formed by *C. albicans* and *Citrobacter freundii*, and also *Trichosporon asahii* combined with *S. simulans*. In both cases, fungi formed the core and bacteria formed the periphery of the biofilm [95]. Secondly, Hoarau et al. investigated the microbiota of patients suffering from CD and determined inter- and intra-kingdom correlations [20]. In contrast to the healthy gut community, microbes can form biofilm-like structures in gut ulcers of IBD patients [297]. Hoarau et al. revealed that, as compared with their non-diseased first-degree relatives, the abundance of the bacteria *S. marcescens* and *E. coli* and the yeast *C. tropicalis* was increased in CD patients. When these pathogenswere combined in vitro in biofilms, the resulting biofilms were thicker and contained more *C. tropicalis* hyphae than the monomicrobial biofilms [20].

#### 2.5.3. *C. albicans* and Staphylococci in Mixed Biofilms

One key feature of mixed-species biofilms formed in vitro by *C. albicans* and *S. epidermidis*, *S. aureus*, or *E. coli* is the enhanced resistance to antimicrobial compounds [83,96,101,102,104,298]. The mechanisms involved in mixed biofilms formed by *C. albicans* and *S. aureus* are well studied. Increased antibiotic resistance of *S. aureus* upon contact with *C. albicans* is independent of hyphae formation and the hypha-associated adhesins Als and Hwp [299]. Interestingly, farnesol, a QS molecule produced by *C. albicans* in biofilms, was found to have a long-term effect on *S. aureus* if added solely. Farnesol induced the expression of efflux pumps and thereby increased tolerance of *S. aureus* towards antimicrobial compounds [83]. In vitro, not only the mass of mixed biofilms composed of *C. albicans* and *S. aureus* or *S. epidermidis* was increased, also the three-dimensional structure was altered as compared to single-species biofilms [101,105]. As a result, diffusion of drugs into the mixed biofilm was shown to be reduced, which contributed to the increased antibiotic tolerance of *S. aureus* in this in vitro set-up [102]. Kong et al. demonstrated further that β-1, 3-glucan produced by *C. albicans* as part of the extracellular matrix, coats bacteria, and thereby protects them from antibiotics. This effect was reversible by adding caspofungin to the in vitro biofilm, a compound that inhibits the fungal cell wall enzyme (1→3)-β-d-glucan synthase [102]. Other studies also supported the role of *Candida* extracellular matrix for *S. aureus* and *E. coli* drug tolerance [96,101]. In addition to the expectable effect of caspofungin on the extracellular matrix produced by *C. albicans*, an additional effect of this compound on *S. aureus* was observed by Siala et al. [300]. They analyzed single-species *S. aureus* biofilms in vitro and in vivo using implanted catheters treated with the antifungal caspofungin and found that it increased the activity of the fluoroquinolone antibiotics. According to their study, caspofungin affected the ica operon and thereby altered the *S. aureus* biofilm, resulting in higher permeability for the antibiotics. Similarly, Rogiers et al. showed in their study of mixed *C. albicans* and *S. aureus* biofilms that the antifungal anidulafungin acts synergistically with the antibiotic tigecycline in vitro and in a mouse model for catheter-associated peritonitis [301]. These studies demonstrate that not only direct cross-kingdom interactions between the microbes occur, but that antifungals and antibiotics might also affect each other’s efficiency directly or indirectly.

#### 2.5.4. *C. albicans* and *E. coli* in Mixed Biofilms

In contrast to the synergism of *Candida*–*S. aureus* biofilms, co-culture experiments pairing *E. coli* with *C. albicans* report antagonism during mixed biofilm formation. Secreted factors from *E. coli* significantly impaired biofilm development of different *Candida* species and decreased the formation of hyphae [73,98]. Furthermore, another recent co-culture study demonstrated that *E. coli* kills fungal cells via a soluble factor and magnesium limitation [74]. Although the factor has not yet been characterized, the researchers speculate that it belongs to the bacteriocins, as others showed that *E. coli* can produce different bacteriocins, for example colicins and microcins, which have antimicrobial action [302].

#### 2.5.5. *C. albicans* and Staphylococci during Tissue Invasion and Systemic Infections

In addition to synergism during biofilm formation, *C. albicans* and *S. aureus* also act together during tissue invasion. Staphylococci were shown to bind to *C. albicans* hyphae [65,101,103,104]. This binding was demonstrated in vitro using atomic force microscopy to be mediated by the *Candida* adhesin Als3 and was also observed in Als3-expressing, non-filamentous *S. cerevisiae* [62]. Binding of *S. aureus* to *C. albicans* hyphae promoted bacterial invasion into tissue in an ex vivo mouse tongue infection model and in vivo in an oral co-infection model using immunosuppressed mice [62,63,64]. However, whether or not these interactions depend on Als3 is not fully clear. While Peters et al. and Schlecht et al. reported the *C. albicans*–*S. aureus* synergism to be Als3-dependent, Als3 was dispensable in the oral candidiasis study of Kong et al. Interestingly, the latter described that treating the underlying *Candida* infection with antifungals cross-protected the mice from the progression of the bacterial infection [64]. Als proteins and O-mannosylation are also involved in the binding of *S. pidermidis* to *C. albicans* [65]. Similar to *S. aureus*, *S. epidermidis* infections seem to be supported by *C. albicans*. In a subcutaneous catheter infection model, the presence of *C. albicans* led to increased dissemination of *S. epidermidis* [105]. 

#### 2.5.6. Mixed Bloodstream Infections in Patients

Severe systemic infections originate from surgery and trauma wounds, or biofilms on medical devices such as central venous or bladder catheters or medical implants [303,304,305]. In other cases, bloodstream infections (BSIs) stem from the gut as the most densely colonized organ [306]. Accordingly, microbes from all the sites mentioned above are commonly isolated from blood cultures, where staphylococci and enterococci are the most prominent, followed by *C. albicans*, *P. aeruginosa,* and *E. coli* [307,308]. 

While the exact numbers vary, many studies do also report polymicrobial BSIs. Especially mixed infections with bacteria and fungi can be associated with increased mortality as compared with mono-infections or poly-bacterial BSIs [11,304,308,309,310,311,312]. Unfortunately, simultaneous diagnosis of fungi and bacteria from blood cultures can be challenging, and especially the fungal components often remain undetected [9,310,313,314]. Recent studies estimate that about 5–38% of candidemia cases are mixed BSIs of *Candida* spp. and bacteria [11,12,13,14]. Of note, multi-*Candida* BSIs were also reported [315]. *Candida* spp. isolated from blood in order of frequency are *C. albicans*, *C. glabrata*, *C. tropicalis*, and *C. glabrata* [315]. Bacteria that accompany *Candida* in BSIs most frequently are staphylococci, enterococci, and *Klebsiella* [11,13,304,311]. Bacteria that are often co-isolated with *Candida* spp. from infection sites are staphylococci [10,316], but also less frequent bacteria such as *S. marcescens*, *Tropheryma whipplei* or other fungi, for example, *C. neoformans* [317,318,319].

#### 2.5.7. Mixed Systemic and Bloodstream Infections in Mouse Models

Synergism of *C. albicans* and bacteria also occurs in systemic infections that are not necessarily associated with biofilm formation. Already in the 1980s, Carlson et al. described increased dissemination of bacteria and mortality in mice infected intraperitoneally with *C. albicans* and *S. aureus*, *S. marcenscens*, or *E. faecalis* [320,321]. Of note, some *S. aureus* strains analyzed were less synergistic than others [321]. More recently, the in vivo synergism of *S. aureus* and *C. albicans* during intraperitoneal infection of mice was analyzed in greater detail [322,323]. In mice that were co-infected intra-abdominally and developed symptoms, levels of proinflammatory cytokines and chemokines were increased while the microbial burden remained unaffected [322,324,325]. Enhanced inflammation was at least in part responsible for the increased mortality in co-infections, whereas reducing inflammation by inhibition of prostaglandin 2 signaling improved survival [324]. 

Furthermore, synergistic pathogenesis was independent of filamentation as injecting yeast-locked or hypha-locked *C. albicans* led to the same outcome [323]. It did, however, depend on the presence of both microbes present at the same site, as no mortality was observed if *S. aureus* was injected intraperitoneally and yeast-locked *C. albicans* was injected intravenously [323,325]. The synergistic potential of the *Candida* species in this model differed. High mortality following co-infection with *S. aureus* was observed for *C krusei* and *C. tropicalis*, whereas, little to no mortality was observed with *C. dubliniensis*, *C. parapsilosis*, and *C. glabrata* [325,326]. Interestingly, a recent study by Lilly et al. demonstrated that the intraabdominal co-infection of mice with *S. aureus* and *C. glabrata* or *C. dubliniensis* was not only non-synergistic but also protected mice against re-challenge with *S. aureus* and *C. albicans*. This protection depended on the presence of live *C. dubliniensis* or heat-killed *C. dubliniensis* and live *S. aureus* [326]. The exact mechanism is not yet clear, but the authors provided evidence that trained immunity via neutrophils might be involved [326].

Studies of murine co-infections with *C albicans* and *E. coli* were, in contrast, less consistent in their outcome. Depending on the study, intraperitoneal co-infections were either non-synergistic or synergistic [327,328]. A possible explanation is likely the strong strain variation with the different *E. coli* “pathotypes” [329]. Synergistic interactions between *E. coli* and *C. albicans* have also been observed in mice co-infected intravenously [330]. Here, mortality after co-infections occurred earlier and the fungal burden in kidneys increased. Furthermore, increased serum TNF levels were observed, that could also be induced by injecting only the corresponding *E. coli* LPS together with heat-killed yeasts [330]. This implies that the combination of key pathogen-associated molecular patterns (PAMPs) from bacteria and fungi might be sufficient to induce a faster and more pronounced immune response which contributes to pathogenesis.

In the studies mentioned above, systemic infections were caused by introducing individual microbes or microbe combinations either into the peritoneum or the bloodstream. Another in vivo sepsis model is cecal ligation and puncture (CLP). For this, the cecum of mice is punctured to induce leakage of fecal matter into the abdomen, which results in inflammation. The size and number of punctures can be adjusted, and thereby the level of inflammation controlled [331,332]. In addition, several infection models can be combined to mimic situations in different patients at risk. For instance, David et al. proposed in their study in 2011 that sepsis causes immunosuppression that predisposes towards secondary infection, with for instance *C. albicans*. To analyze this, mice underwent mild CLP followed by intravenous injection of *C. albicans*. Mortality in co-infected mice was increased as compared with mice infected with *C. albicans* only and depended on the timing of secondary infection. The mice were more susceptible shortly after CLP rather than later on when the immune system was partially reconstituted [333]. In line with this, it was shown that monocytes from CLP-treated mice expressed less antifungal effector genes upon secondary intravenous *C. albicans* challenge, had less inflammatory monocytes in circulation, and less neutrophil influx into the kidneys [334]. In mice that received *C. albicans* via oral gavage a few hours after CLP, mortality was also increased as compared with CLP-treated animals [335]. In these mice, increased mortality was accompanied by increased serum (1→3)-β-d-glucan levels and could also be achieved with heat-killed fungal cells [335]. Already, within a short amount of time, introduction of *C. albicans* altered the microbiota. The abundance of *Bacteroides* was increased while the abundance of *Lactobacillus* was decreased [335]. 

Similarly, (1→3)-β-d-glucan was increased in serum of mice that received *C. albicans* first as oral gavage followed by antibiosis for several days before CLP was performed. Of note, in this setup no candidemia was detectable. Nevertheless, mortality, as well as serum IL-6 concentration, were affected by the dose of the *Candida* inoculum [336]. In addition to CLP, other strategies exist to recreate a leaky gut. When CLP and treatment with LPS were compared side-by-side, two out of three mouse models of gut disintegration led to rapidly increased serum (1→3)-β-d-glucan levels: DSS-treatment, a well-known colitis model, was less potent [337]. When sera from septic patients, both fungal and bacterial, were analyzed, (1→3)-β-d-glucan correlated with sepsis severity and increased IL-6 levels were associated with gut leakage and (1→3)-β-d-glucan [337]. The results from both of these studies illustrate the potential of fungal (1→3)-β-d-glucan to be a marker for both bacterial and fungal sepsis [338]. Nevertheless, it should be mentioned that glucans by themselves are also immunomodulatory as they bind to PRRs [339]. Taken together, these studies of systemic infections demonstrate that certain combinations of bacteria and fungi significantly alter morbidity and mortality. This appears to be driven to a large extent by the response of the immune system to the simultaneous presence of bacterial and fungal PAMPs and suggests a major role of the immune response for pathogenesis of co-infections.

## 3. Conclusions

Over the last years, the impact of microbial interactions in health and disease has been increasingly recognized, and consequently, many researchers have left the historical separation of pro- and eukaryotes behind and started investigating co-colonization and co-infections beyond kingdom barriers.

One major challenge that researchers are facing today is the translation of findings from one experimental or investigative approach to the other and eventually into the clinical setting.

In many cases, health and disease associations of certain fungal-bacterial combinations identified in studies of complex microbiomes are the starting point for investigations. Those are then converted to less complex experimental set-ups with only a handful of microorganisms in order to study them explicitly. Here, a typical approach is to study interactions of distinct species using co-cultures in rich media, co-infections in cell cultures, invertebrates, and vertebrates such as mice or fish. This so-called reductionist approach has provided valuable insights into the nature of certain fungal-bacterial interactions. However, this approach also has its limitations.

In comparison to the high number of fungal and bacterial species in the human body only a small selection has been studied so far regarding their cross-kingdom interplay. By this, the species of interest are stripped of their natural microbial habitat and researchers might also have missed interesting combinations. Furthermore, variations within bacterial and fungal strains are often not investigated due to technical or practical limitations.

To overcome these issues, experimental set-ups and models need to be increased in complexity. Instead of using one fungal and one bacterial strain during the initiating experiments, several of each could be included. Instead of using cell cultures, organoids or organ-on-chip models could be used. Instead of using in vitro models, germ-free animals could be used to test certain fungal-bacterial combinations in vivo. And finally, instead of using mono- and co-colonized animals, reduced floras with a defined composition of several microbes could be used.

Interactions between microbes are not only affected by the specific combination of microbes, but also by the environment. When translated to the human host, different anatomical niches have distinct nutritional and immunological properties, which in return are likely to affect the bacterial-fungal interplay. While some constellations of fungus and bacterium might be neutral or even beneficial in a healthy human in one niche, the exact same duo could be devastating in a critically-ill immunocompromised patient in another niche. These limitations need to be considered when findings from fungal-bacterial interaction studies are translated from one setup to another.

In summary, it is the triangle of fungi, bacteria, and host that shapes the behavior of microbes and the overall outcome of their interplay for the host. As a basic principle, high diversity communities seem to be beneficial for the host while low diversity communities bear higher risks (Figure 1), and the future challenge will be to understand these interactions on both the molecular level and in their complexity. To meet this challenge, improved approaches and collaborations among bacteriologists, mycologists, immunologists, and clinicians are required which could provide the foundation for personalized microbiology. Then, a deeper understanding of the fungi–bacteria–host triangle might allow identification of patients who are at risk and improvement of patient care by tailored manipulation of the microbiota.

Box 1 **Short introduction of key microbes (in alphabetic order).**

**Aspergilli** are saprophytic molds, growing primarily on rotting biological material from where they spread via air as small and light spores. Unlike *Candida* spp., *Aspergillus* spp. are no natural colonizers of the human body. After inhalation, conidia can cause allergic reactions or severe diseases, like chronic pulmonary infections in patients with an impaired immune system. Major infective agents are *A. fumigatus* and *A. nidulans.* During infection, conidia swell, form germlings, and eventually long filaments [98,340,341].
***Candida albicans*** is an opportunistic fungal pathogen that causes disease mostly in immunocompromised patients [342,343]. In healthy individuals, its major reservoir is the gut, but this yeast can be found in many niches of the human body, for instance throughout the entire GI tract [344,345]. The most relevant virulence trait of *C. albicans* is the ability to switch from yeast to hypha form and thereby either proliferate or adhere, penetrate tissues, and disseminate [346].
**Enterococci** are opportunistic Gram-positive lactic acid producing bacteria commonly found as members of the microbiota of mammals and in a wide range of environmental niches. This wide distribution is due to their high tolerance against pH extremes, elevated temperatures, as well as salt concentrations. In humans, *E. faecalis* and *E. faecium* cause nosocomial infections like UTIs, bacteremia, and endocarditis [347].
***Escherichia coli*** is a Gram-negative bacterium that is the most prominent cause of infections in humans. As a common colonizer of the gut, it is also a widely-used indicator of fecal contaminations in food or water. *E. coli* shows a unique pathovariety from probiotic to life-threatening [329]. For instance, enterotoxigenic *E. coli* (ETEC), which cause moderate-to-severe diarrhea and can lead to malnutrition and death in young children, is one of the main health problems in developing countries [348].
***Klebsiella pneumoniae*** is a Gram-negative bacterium that can be found in the lung and throughout the human GI tract. Mostly harmless, it can cause a variety of infections. Especially nosocomially-acquired pneumonia can be problematic for immunocompromised patients in the hospital setting. *K. pneumoniae* possesses a thick capsule that protects it from the host immune system and other external threats and leads to characteristic slimy colonies.
***Lactobacillus* spp**. are Gram-positive, facultatively anaerobic bacteria that are also part of the gastrointestinal and vaginal flora [349]. They belong to a group of lactic acid producing bacteria (LAB) which ferment carbohydrates and produce lactic acid. Other genera of this group are, for example, *Streptococcus*, *Lactococcus*, and *Enterococcus* [349,350].
***Mucorales*** are ubiquitous molds that are able to cause infections opportunistically in immunocompromised and diabetic individuals, called mucormycosis [288,289,290]. The infections, so-called mucormycoses, are typically acquired via spores and filaments are formed within the infected organs. Mucormycosis often originates in the respiratory tract, but commonly disseminates into other organ systems [288].
***Pseudomonas aeruginosa*** is a Gram-negative bacterium that is well-adapted to many different niches. It survives in the environment but is also able to cause severe infections in humans. Especially in CF-patients, *P. aeruginosa* is feared for its ability to cause persistent lung infections. The ability of *P. aeruginosa* to form biofilms is of special importance in its virulence [351,352].
***Serratia marcescens*** is a Gram-negative opportunistic pathogen that forms characteristic red colonies when grown on culture media. It causes mainly UTIs, but also wound and bloodstream infections, mostly in neonates. It tolerates temperatures at 5–40 °C, is found ubiquitously in the environment, and is among the top ten isolated pathogens from hospitals all over the world [353,354].
**Staphylococci** are Gram-positive bacteria. The two most relevant species in human disease are the coagulase-negative *S. epidermidis* and the coagulase-positive *S. aureus*. As commensals, *S. epidermidis* is a frequent colonizer of human skin whereas *S. aureus* can be found in the nasal cavities of roughly 30% of the human population. Staphylococci can cause a wide range of infections, from superficial to systemic and life-threatening [355,356].
**Streptococci** are Gram-positive bacteria that include a wide range of opportunistic pathogens with various virulence factors. *S. pyogenes* (Group A Streptococci) is the causative agent of diseases such as scarlet fever, impetigo, and necrotizing fasciitis. *S. pneumoniae* (pneumococci) causes infections of the respiratory tract. Oral streptococci (group *viridans*) can cause local infections of oral mucosa and caries. Especially malignant are long-term complications such as endocarditis due to crossreactive antibodies produced during acute infection [357,358,359].

## Glossary

Polymicrobial	Referring to more than one microbe; can be poly-fungal, poly-bacterial, or fungal-bacterial
Monoinfections	Infections with one microbe; either fungal or bacterial
Co-infections	Infections with more than one microbe; can be poly-fungal, poly-bacterial, or fungal-bacterial
Mixed biofilms or infections	Biofilms or infections with at least one bacterium and one fungus
Microbiota/microbiome	Entity of bacteria/bacterial genes in a certain niche or sample
Mycobiota/mycobiome	Entity of fungi/fungal genes in a certain niche or sample
Microbes/microbial:	In general, referring to bacteria and fungi
